# Improving gait adaptability in patients with hereditary spastic paraplegia (Move-HSP): study protocol for a randomized controlled trial

**DOI:** 10.1186/s13063-020-04932-9

**Published:** 2021-01-07

**Authors:** Lotte van de Venis, Bart P. C. van de Warrenburg, Vivian Weerdesteyn, Bas J. H. van Lith, Alexander C. H. Geurts, Jorik Nonnekes

**Affiliations:** 1 Department of Rehabilitation; Center of Expertise for Parkinson & Movement Disorders, Donders Institute for Brain, Cognition and Behavior; Radboud University Medical Center, PO Box 9101, 6500 HB Nijmegen, The Netherlands; 2 Department of Neurology; Center of Expertise for Parkinson & Movement Disorders, Donders Institute for Brain, Cognition and Behavior; Radboud University Medical Center, Nijmegen, The Netherlands; 3 Department of Rehabilitation, Sint Maartenskliniek, Ubbergen, The Netherlands

**Keywords:** Hereditary spastic paraplegia, C-Mill, Gait adaptability, Rehabilitation

## Abstract

**Background:**

People with hereditary spastic paraplegia (HSP) experience difficulties adapting their gait to meet environmental demands, a skill required for safe and independent ambulation. Gait adaptability training is possible on the C-Mill, a treadmill equipped with augmented reality, enabling visual projections to serve as stepping targets or obstacles. It is unknown whether gait adaptability can be trained in people with HSP.

**Aim:**

The aim of Move-HSP is to study the effects of ten 1-h sessions of C-Mill training, compared with usual care, on gait adaptability in people with pure HSP. In addition, this study aims to identify key determinants of C-Mill training efficacy in people with pure HSP.

**Method:**

Move-HSP is a 5-week, two-armed, open-label randomized controlled trial with a cross-over design for the control group. Thirty-six participants with pure HSP will be included. After signing informed consent, participants are randomized (1:1) to intervention or control group. All participants register (near) falls for 15 weeks, followed by the first assessment (week 16), and, thereafter, wear an Activ8 activity monitor for 7 days (week 16). The intervention group receives 10 sessions of C-Mill training (twice per week, 1-h sessions; weeks 17–21), whereas control group continues with usual care (weeks 17–21). Afterwards, both groups are re-assessed (week 22). Subsequently, the intervention group enter follow-up, whereas control group receives 10 sessions of C-Mill training (weeks 23–27), is re-assessed (week 28), and enters follow-up. During follow-up, both groups wear Activ8 activity monitors for 7 days (intervention group: week 23, control group: week 29) and register (near) falls for 15 weeks (intervention group: weeks 23–37, control group: weeks 29–43), before the final assessment (intervention group: week 38, control group: week 44). The primary outcome is the obstacle subtask of the Emory Functional Ambulation Profile. Secondary outcomes consist of clinical tests assessing balance and walking capacity, physical activity, and fall monitoring.

**Discussion:**

Move-HSP will be the first RCT to assess the effects of C-Mill gait adaptability training in people with pure HSP. It will provide proof of concept for the efficacy of gait adaptability training in people with pure HSP.

**Trial registration:**

Clinicaltrials.gov NCT04180098. Registered on November 27, 2019.

**Supplementary information:**

**Supplementary information** accompanies this paper at 10.1186/s13063-020-04932-9.

## Background

Hereditary spastic paraplegia (HSP) is a heterogeneous group of neurodegenerative disorders, caused by retrograde axonal degeneration of the corticospinal tracts, fasciculus gracilis fibers, and to a lesser extent, the spinocerebellar fibers [1–3]. Pure forms of HSP are clinically characterized by progressive spasticity, muscle weakness, and reduced proprioception in the lower extremities, as well as difficulties in making rapid (alternating) leg movements [4–6]. Additional symptoms are present in complex forms of HSP, including mental retardation, epilepsy, ataxia, peripheral neuropathy, or optic atrophy [1, 4, 7]. For people with pure HSP, gait and balance impairments are among the most disabling symptoms. They especially experience difficulties when forced to adapt their gait to meet environmental demands, hampering the ability to walk safely and independently in the community [4, 8–11]. A recent study reported that 57% of pure HSP patients fell at least twice a year, and 73% experience fear of falling [11]. Incorporating gait adaptability training in rehabilitation programs for people with pure HSP seems, therefore, logical and potentially beneficial [4, 11, 12].

A limited number of task-specific gait interventions have shown to improve walking capacity in people with pure HSP. Twenty-five sessions of robot-assisted exoskeleton and overground walking improved walking velocity and balance capacity [13]. In addition, eighteen sessions of robotic Lokomat training increased walking speed, balance capacity, and quality of life [9]. Even though these results are promising, the interventions lacked tasks that promote gait adaptability. As a consequence, it remains unknown whether people with pure HSP will benefit from gait adaptability training [4]. Furthermore, it is unclear how to tailor gait rehabilitation programs to the individual patient with HSP as it is currently unknown which determinants can predict training efficacy.

To fill this gap, Move-HSP is the first randomized controlled trial to provide proof of concept for the efficacy of gait adaptability training in people with pure HSP. The training takes place in a safe environment on the C-Mill, a treadmill providing augmented reality via visual projections onto the treadmill. Participants will train obstacle negotiation, precision stepping, and unexpected accelerations and decelerations. Its feasibility and efficacy have been described in multiple neurological populations, including patients with stroke [14], cerebellar ataxia [15], and multiple sclerosis [16]. Currently, the clinical experience with gait adaptability C-Mill training for people with pure HSP is positive, but the scientific evidence is lacking [4].

### Objectives

This study aims to provide an essential step towards evidence-based and individually tailored gait rehabilitation in people with HSP. The objectives are twofold:

1. To study the effect of ten 1-h sessions of C-Mill training on gait adaptability in people with pure HSP.
2. To identify key determinants of C-Mill training efficacy in people with pure HSP.

## Methods/design

### Regulation statement

Move-HSP will be conducted according to the principles of the Declaration of Helsinki (64th WMA General Assembly, Fortaleza, Brazil, October 2013) and the Medical Research Involving Human Subjects Act. The protocol is written in accordance with the SPIRIT 2013 checklist.

### Study design and setting

Move-HSP is a 5-week, single-center, two-armed, open-label, randomized controlled trial (RCT), with a cross-over design for the control group, as they receive the intervention after a waiting list period. The study is conducted at the Radboud University Medical Center (Radboudumc) within the Center of Expertise for Parkinson & Movement Disorders; Nijmegen, The Netherlands. C-Mill training can be given at the Radboudumc (Nijmegen, The Netherlands), Paramedisch centrum Rembrandt (Veenendaal, The Netherlands), Stichting Tante Louise (Bergen op Zoom, The Netherlands), and Fysiotherapie De Lindehoeve (Voorschoten, The Netherlands). Other training locations may be added while the study is ongoing, depending on the success of participant inclusion.

### Recruitment and selection

Participants will be recruited at the Center of Expertise for Parkinson & Movement Disorders of the Radboudumc (part of the European Reference Network for Rare Neurological Diseases (ERN-RND)). The treating physician informs the patient about Move-HSP and asks for permission whether the investigator (LV) may contact the patient. In addition, a request to participate will be sent to members of the HSP patient organization “Spierziekten Nederland”. Those who are interested can contact the investigator and will receive an information letter. After 2 weeks, the investigator (LV) will contact those who expressed their interest and ask for their final decision. If patients agree to participate, eligibility is checked. After inclusion, participants can leave the study at any time without consequences.

### Eligibility

For inclusion, participants will have to meet the following inclusion criteria:

- Diagnosis of pure HSP by a neurologist specialized in inherited movement disorders. Diagnosis is based on inheritance pattern and clinical examination, and when available, molecular diagnosis.
- Age between 18 and 70 years old
- Ability to walk barefoot on a level ground for 50 m without a walking aid (use of orthotic devices or orthopedic shoes is allowed)

Participants will be excluded if they suffer from other neurological, orthopedic, or psychiatric conditions, or if patients underwent an HSP-related surgical procedure of the lower extremities.

### Group allocation and blinding

Participants will be allocated at random to the intervention group or to the (waiting list) control group following a 1:1 ratio. Randomization will be stratified based on disease duration (2 categories: 0–15 years; > 15 years) in blocks with a variable size (*n* = 4 or *n* = 6) to prevent an uneven distribution between groups. To determine disease duration, participants are asked for the year of symptom onset. Randomization will be performed in CastorEDC, a web-based data management system for academic studies (www.castoredc.com). Blinding of participants is not possible, as participants will know whether they receive C-Mill training or continue with usual care. The primary investigator (LV) takes part in the training sessions as a physical therapist and, therefore, cannot be blinded either.

### Participant timeline

**Fig. 1:**
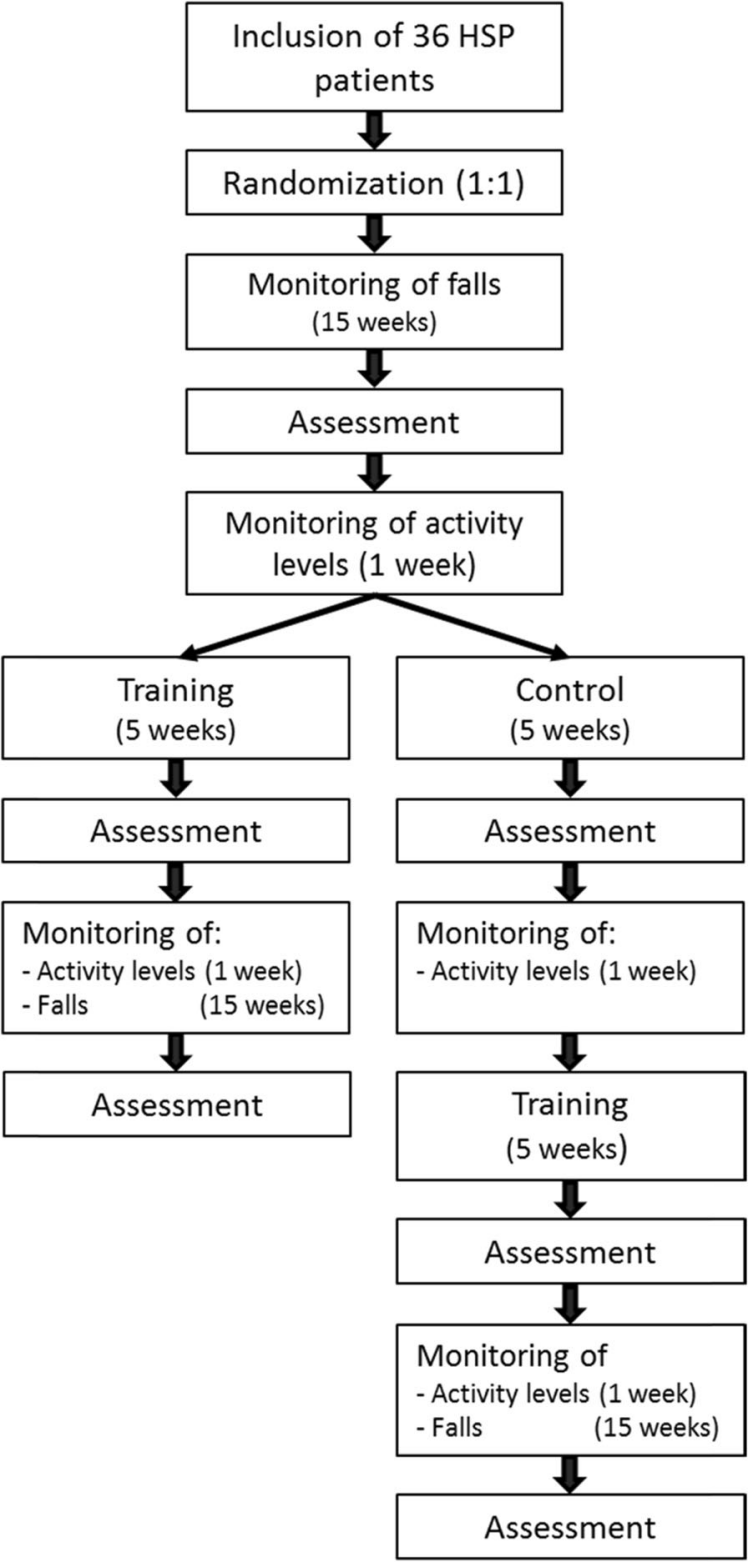
Flowchart of the study protocol

The outline of this study is shown in Fig. 1. Following inclusion, participants are randomly allocated to either the intervention group or the control group (waiting list). During the first 15 weeks, all participants register (near) falls in a digital fall calendar. Thereafter, participants will have the first assessment at the movement laboratory (Radboudumc; week 16). Following this assessment, participants wear an Activ8 activity monitor for seven consecutive days (week 16). Thereafter, the *control group* enters a waiting period of 5 weeks (weeks 17–21), whereas the *intervention group* starts with 5 weeks of gait adaptability training on the C-Mill (weeks 17–21). Each session lasts 1 h and takes place twice per week. Subsequently, both groups are re-assessed (week 22). Following this second assessment, the intervention group enters the follow-up period, whereas the control group wears the Activ8 activity monitors for 7 days (week 22), starts 5 weeks of gait adaptability training (weeks 23–27), has the third assessment (week 28), and thereafter, enters the follow-up period. During follow-up, both groups wear Activ8 activity monitors during the first week (intervention group: week 23, control group: week 29) and, additionally, register (near) falls for 15 weeks (intervention group: weeks 23–37, control group: weeks 29–43). After follow-up, participants have a final assessment in the movement laboratory (intervention group: week 38, control group: week 44).

During Move-HSP, all participants can continue their usual care. For some participants, this may include local intramuscular injections of botulinum toxin (BTX). To limit the influence of BTX injections on the outcomes, the scheduling of the assessments will consider the date of the BTX injections. BTX injections induce an effect on muscle spasticity approximately 2 weeks post-injection. The maximum effect is reached around 6–8 weeks, after which it gradually subsides [17, 18]. Participants who receive BTX injections in the lower extremities will have the pre-intervention assessment 4 weeks post-injection, and the post-intervention assessment 10 weeks post-injection. In addition, it will be monitored whether the dosage of oral antispasmodic changes during the trial.

### Control group

The eighteen participants attributed to the control group are asked to continue with their daily routine and usual care during the 5 weeks on the waiting list. If therapy is part of the usual care, participants are requested to continue with the same frequency and composition during the waiting period.

### Intervention: C-Mill training

Gait adaptability training takes place on the C-Mill (Motek Medical, Culemborg, The Netherlands). The C-Mill is a treadmill, providing augmented reality via visual cues projected onto the treadmill. The projections are either stepping targets or obstacles that challenge the participants to adjust their steps accordingly. The training sessions take place during five consecutive weeks, twice per week during 60-min sessions. In total, participants will train gait adaptability on the C-Mill for 10 h.

**Fig. 2:**
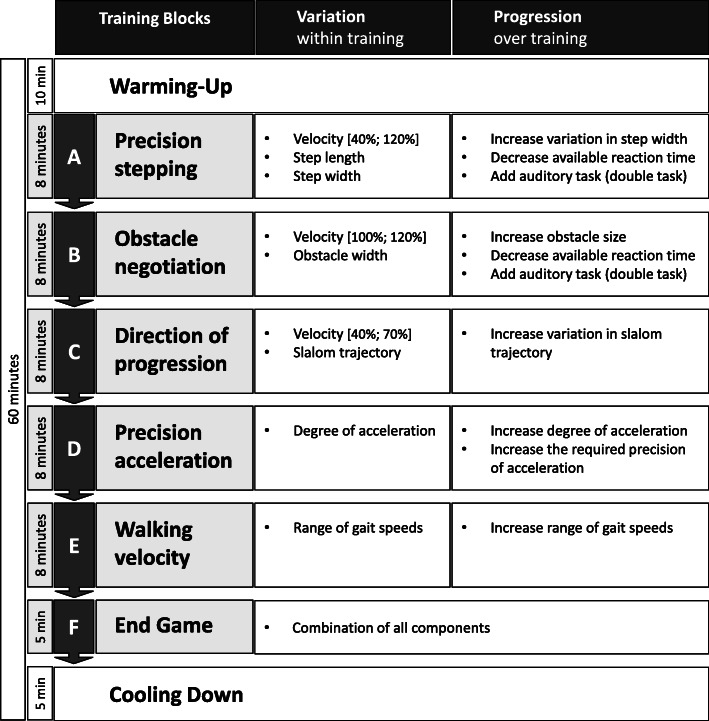
Overview of the C-Mill training

The C-Mill protocol is based on clinical experience and finalized after a focus group discussion with expert physical therapists. The training sessions are logged to ensure compatibility and a consistent progression. Each session starts with a 10-min warming-up, followed by five training blocks (Fig. 2, video). Each training block lasts approximately 8 min. Block A targets precision stepping by practicing accurate foot placement on the projected stepping tiles. Block B targets obstacle negotiation by avoiding the projected obstacles. Block C elicits changes in the direction of progression by using a variety of slalom trajectories. Block D targets precision acceleration and deceleration, as the participants must walk within a projected square that moves forward and backward on the treadmill. Block E challenges walking at different walking speeds. Block F is the endgame, a 5-min track that combines several gait adaptability components in an interactive way. All sessions end with a cooling-down. To further promote the level of variability, each training block consists of small components (i.e., for block A: Stepping Tiles: belt speed will momentarily increase; width between the stepping stones will momentarily decrease). In addition, different walking speeds are used: 100% is the participant’s comfortable walking speed on the treadmill. This will be determined during the first training session. The belt speed will be manually increased until the participant experiences it as comfortable. The therapist will then increase the belt speed with 0.3 m/s and slowly decrease the belt speed until the participant again experiences it as comfortable. The average of both speeds will be used to set the comfortable walking speed. Other percentages (e.g., 40%, 70%, 120%) are derived from this reference speed. The C-Mill training will be carried out by a physiotherapist with C-Mill certification. Progression over the training period is initiated and controlled by this therapist and based on the patient’s capacity and performance. It comprises of increasing the level of task variability, increasing obstacle size, and the addition of a dual task, for example the use of the auditory Stroop task. During the Stroop task, participants listen to an audiotape presenting a random sequence of the words “high” or “low”, expressed in either a low-pitched voice or a high-pitched voice. They are asked to respond aloud indicating the pitch of the word (“high” or “low”), while ignoring the (randomly conflicting) semantic meaning of the word.

### Procedure and assessments

All outcome measurements will be collected during the assessments at the movement laboratory (Table 1). The intervention group is evaluated three times: pre C-Mill training (week 16), post C-Mill training (week 22), and at the end of the follow-up (week 38). The control group is evaluated four times: pre waiting-list (week 16), post waiting-list (week 22), post C-Mill training (week 28), and at the end of the follow-up (week 44). The assessments will follow a standardized protocol and are conducted by the primary investigator (LV) who is trained to perform the outcome measurements. As the primary investigator takes part in the training sessions, outcome assessment cannot be blinded. During all assessments, the use of orthotic devices and/or orthopedic footwear is allowed depending on the task. No other (walking) aids are allowed. If participants use any orthotic or orthopedic device during a task, this will be registered and kept constant throughout the consecutive assessments.

**Table 1:**
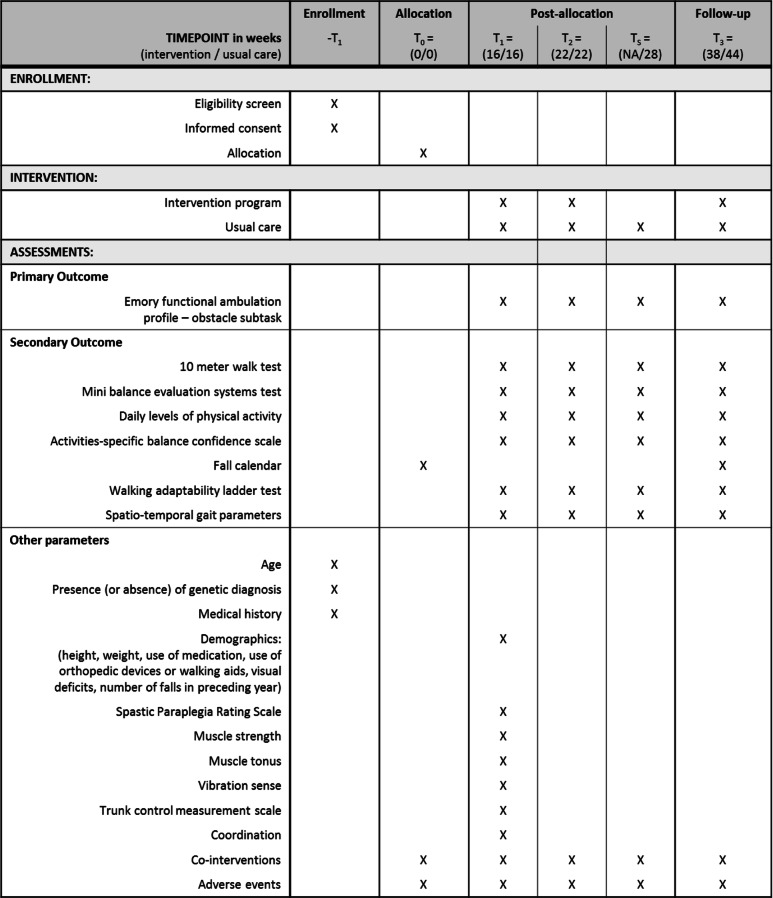
Standard protocol items: Recommendations for interventional trials (SPIRIT) figure

Overview of enrollment, interventions, and assessments during the study protocol. Timepoints T_1_, T_2_, and T_3_ are applicable for participants in the intervention group and usual care group. Timepoint T_s_ is only applicable for participants in the usual care group

*NA* not applicable

### Demographic and clinical assessments

The demographic and clinical assessments are collected during the first assessment in the movement laboratory. The demographic characteristics consist of age; sex; height; weight; presence (or absence) of a genetic diagnosis and inheritance pattern; disease duration (i.e., the number of years since symptom onset); regular use of medication, orthopedic shoes or orthotic devices, or other walking aids; presence and severity of visual deficits; and number of falls in the preceding year.

Clinical assessments consist of the Spastic Paraplegia Rating Scale (SPRS) to determine disease severity [19]. Bilateral muscle strength is scored with the Medical Research Council (MRC) scale for hip adduction, abduction, flexion, and extension; knee flexion and extension; and ankle plantar and dorsiflexion [4, 20, 21]. Bilateral muscle tonus is scored with the Modified Ashworth Scale (MAS) for the hip adductors (hip 70° flexed), knee flexion and extension, and ankle plantar and dorsiflexion with knee extended (gastrocnemius) and knee flexed (soleus) [22]. Vibration sense is evaluated using a tuning fork on bilateral patella, lateral malleolus, and at the first metatarsophalangeal joint. Trunk control is assessed using the Trunk Control Measurement Scale (TCMS) [23]. Lastly, coordination is examined via (i) toe tapping, and (ii) leg agility [24].

### Primary outcome

The primary outcome is gait adaptability as measured with the obstacle subtask of the Emory Functional Ambulation Profile (E-FAP). Participants are asked to negotiate a 10-m course in which two wooden blocks (100(l) × 10(w) × 5(h) cm) and a bin are placed along the walkway. The instruction given is to complete the task as fast as possible but keep your own safety in mind. The number of seconds needed to complete the task is registered. A faster time score indicates better gait adaptability. The obstacle subtask of the E-FAP has previously been used as an outcome measure for gait adaptability in several neurological populations [14, 15]. The full protocol is available online and via Wolf et al. [25].

### Secondary outcomes

Secondary outcome measures comprise of the following clinical tests:

- 10-Meter Walk Test (10MWT)

The *10MWT* is a standardized and recommended measurement of walking velocity. Participants walk 13 m in a straight line, first three times at comfortable speed and then three times as fast as possible. Participants have 3 m to accelerate to the requested speed. When the first foot crosses the 3-m line, the timer starts. The timer stops when the first foot crosses the 13-m line. Like this, the number of seconds it takes to walk 10 m is recorded [26]. The test has been found reliable, valid, and sensitive in neurological populations [26] and has been used in people with HSP [9, 13, 27, 28].

- Mini Balance Evaluation Systems Test (miniBEST)

The *mini-BEST* is a 14-item, 3-point ordinal rating scale (0–2 points) to evaluate balance capacity in 4 subcategories: anticipatory postural control, reactive postural control, sensory orientation, and gait stability. The attainable sum scores range from 0 to 28 points, a higher score indicating better balance capacity. Participants perform the test barefoot. The full protocol is available online and has been described by Franchignoni et al. [29]. The mini-BEST is often used in neurological populations; has been found valid, reliable, and responsive [30–32]; and has been recommended for use in people with HSP [4].

- Physical activity levels during daily life

*Physical activity* during daily life will be registered via the Activ8 Physical Activity Monitor (Activ8, Remedy Distribution Ltd., Valkenswaard, The Netherlands). The Activ8 monitor is a small (30 × 32 × 10mm) and lightweight device with three axial accelerometers. It registers body positions (non-wear of the Activ8/lying, sitting, and standing) and activities (walking, running, cycling) [33]. The Activ8 is placed by the primary researcher using Tegaderm™ tape on the right upper thigh of the participants. Interval for data sampling will be set to one measurement per 15 s. Collected measures consist of total time spent walking (minutes) and total time spent active (i.e., minutes classified as walking, running or cycling).

- Activities-specific Balance Confidence scale (ABC)

*Balance confidence* will be measured using the ABC. The questionnaire describes sixteen indoor and outdoor situations. Participants are asked to express their confidence in safely executing the proposed situations without falling. Scores range from 0 to 100, a higher score indicating more confidence. The ABC has been used to assess balance confidence in people with HSP [27, 34]. The full questionnaire is available via Powell et al. [35].

- Fall calendar

The *fall calendar* is used to monitor falls and near falls and is self-reported by the participants during a 15-week period. The World Health Organization defined a fall as “an event which results in a person coming to rest inadvertently on the ground or other lower level.” A near fall is defined as “a stumble event or loss of balance that would result in a fall if sufficient recovery mechanisms were not activated” [36]. In addition, participants register incidents where a fall was likely to happen, but was averted through the action of another person. When a (near) fall occurs, the participant is asked to report a short description of the event, the environment (indoor/outdoor, illuminated/dark space, and surface (e.g., tiles, carpet, forest)), and lastly, whether the (near) fall resulted in any injuries. To meet participants’ preference, calendars can be filled in digitally or on paper. Every other week, participants are reminded of the fall calendar via a phone call from the primary investigator (LV).

- Walking Adaptability Ladder Test (WALT)

The *Walking Adaptability Ladder Test (WALT)* is a test to measure step precision. A standardized ladder is placed on the floor. It consists of 17 rectangular stepping targets that gradually decrease 2 cm in length (range 64–32 cm). Participants start stepping in the largest target and walk as fast as possible to the other side, turn and hit the targets in reverse order while avoiding the ladder rungs. The instructions are to perform the test as fast as possible, but try to prevent foot placement errors. The test is timed: a faster time is indicative of better stepping precision. Participants perform the test four times, first twice with one foot per target and, thereafter, twice with both feet in one target. A time penalty of 0.5 s is added each time a participant makes a foot placement mistake.

- Spatio-temporal gait parameters

*Spatio-temporal gait parameters* are collected with a 3D full body gait analysis using Vicon (Vicon© Motion systems Ltd.) at the movement laboratory of the Radboudumc, Nijmegen. Retroreflective markers are placed on anatomical landmarks according to the standard Plug-in Gait marker model for upper and lower body. In addition, participants will wear accelerometers on their lateral heels, as the higher measuring frequency will enable a more accurate gait event detection. Participants will walk two bouts of 3 min over an 8-m walkway. The following spatio-temporal parameters will be retrieved: step length (cm), step width (cm), step time (s), walking speed (m/s), stride time (s), stride length (cm), and cadence (steps/min).

### Assessment of therapy adherence and co-interventions

To support adherence to the protocol, participants will be in direct contact with the primary investigator (LV) by telephone every other week. This enables the investigator to verbally confirm assessments and training dates, check adherence to the fall calendar, and quickly address and resolve questions and possible problems that may interfere with continuation of the protocol. In addition, participants are offered flexible time slots for the assessments and training sessions. Assessment of adherence to the C-Mill protocol is possible as therapists will log the performed C-Mill trainings. In case of an unexpected cancelation, the reason will be registered, and the missed training can be compensated in the next week. When multiple consecutive training sessions cannot proceed, a pragmatic solution is sought so that the participant is able to complete the protocol.

In addition, assessment of co-intervention will take place during the assessments. Participants are asked to self-report in a survey what type of co-intervention they received (e.g., physical therapy, occupation therapy).

### Sample size

Sample size calculation is based on previous studies assessing effectiveness of C-Mill training on the obstacle subtask of the E-FAP scores in neurological populations [14, 15]. A total of 32 participants is sufficient to demonstrate an improvement on the E-FAP score of 1.75 s (SD = 2.0 s, *α* = 0.05, *β* = 0.2). Considering a 10% attrition rate, 36 participants will be included.

### Statistical analysis

The effect of gait adaptability training on primary and secondary outcomes will be tested using ANCOVA. Post-intervention measurements will be used as dependent variables with pre-intervention measurements as the covariate. Group (C-Mill versus waiting list) is used as an independent between-subjects factors. The retention of gait adaptability training will be tested by merging both groups and using a repeated measures ANOVA with time as a within-subjects factor (C-Mill group: assessments 1, 2, and 3; waiting list: assessments 2, 3, and 4). Post hoc tests will be performed in the case of significant main or interaction effects, using paired *t* tests. Fall rates will be processed descriptively. Depending on the distribution of the data, the rate of near falls may be analyzed using Wilcoxon signed rank test. In addition, to determine key determinants of C-Mill training efficacy, a stepwise linear regression analysis will be performed with training-induced change in gait adaptability (relative change of the obstacle subtask of the E-FAP) as the dependent variable. Univariate analyses will be performed to select the best factors from the available demographic and clinical characteristics.

## Discussion

Limitations in walking capacity are among the most disabling symptoms in people with hereditary spastic paraplegia (HSP). A handful of studies aimed to improve walking capacity in people with HSP [9, 13, 37], but these studies did not include context-specific exercises aimed at gait adaptability. Gait adaptability has been successfully trained in several neurological populations using augmented reality on the C-Mill [14, 15], but so far, this has never been done in people with HSP.

Move-HSP is a two-armed, open-label randomized controlled trial that will be the first study to assess the effects of gait adaptability training in people with pure HSP. Participants in the intervention group receive 10 h (1-h sessions; twice per week) of protocolled C-Mill training, whereas the control group continues with treatment as usual (waiting list). After 5 weeks on the waiting list, the control group will cross over and follow gait-adaptability training. The primary outcome is gait adaptability assessed with the obstacle subtask of the E-FAP. Secondary outcomes focus on several aspects of balance and gait capacity. Mildly to moderately affected people with pure HSP that fit the a-priori established eligibility criteria will be included. There are no restrictions regarding sex, symptom duration, or use of orthotic/orthopedic devices in order to represent the clinical heterogeneity characteristic of people with pure HSP. Yet, to provide proof op principle and limit the influence of impaired cognitive capacity, people with complex forms of HSP are excluded.

Move-HSP aims to make a step towards evidence-based and individually tailored gait rehabilitation programs for people with HSP. It will reveal whether context-specific training is an effective tool for improving gait adaptability in people with pure HSP. If the C-Mill intervention results in improved walking adaptability, it may be beneficial to implement this type of training on a regular basis in the rehabilitation of people with HSP. In addition, knowledge of the key determinants of training efficacy will help to optimize the selection of subjects with HSP that are most responsive to gait adaptability training.

### Trial status

Participant recruitment has started in November 2019 and is currently ongoing. So far, no assessments in the movement laboratory have yet been conducted. It was originally anticipated that a total of 10 months would be needed to complete the recruitment and 24 months to complete the entire protocol, but the current SARS-CoV-2 pandemic will undoubtedly lengthen these periods to an unforeseeable extent. This study protocol is based on protocol version 5, dated October 31, 2019. An amendment to add training locations was approved on February 18, 2020.

## Supplementary information

**Additional file 1.** (MP4 9073 kb)

## Data Availability

Not applicable.

## Abbreviations

10MWT: 10 Meter Walk Test
ABC: Activities-specific Balance Confidence Scale
BTX: Botulinum toxin
E-FAP: Emory Functional Ambulation Profile
ERN-RND: European Reference Network for Rare Neurological Diseases
HSP: Hereditary spastic paraplegia
MAS: Modified Ashworth Scale
miniBEST: Mini Balance Evaluation Systems Test
MRC: Medical Research Council
Radboudumc: Radboud University Medical Center
SPRS: Spastic Paraplegia Rating Scale
TCMS: Trunk Control Measurement Scale
WALT: Walking Adaptability Ladder Test
